# *Pseudomonas aeruginosa lasR*-deficient mutant contributes to bacterial virulence through enhancing the PhoB-mediated pathway in response to host environment

**DOI:** 10.1128/mbio.01788-25

**Published:** 2025-09-04

**Authors:** Xiaolei Pan, Liwen Yin, Dandan Zhou, Yongxin Jin, Zhihui Cheng, Un-Hwan Ha, Shouguang Jin, Weihui Wu

**Affiliations:** 1 State Key Laboratory of Medicinal Chemical Biology, Key Laboratory of Molecular Microbiology and Technology of the Ministry of Education, Department of Microbiology, College of Life Sciences, Nankai University 616162, Tianjin, China; 2 Department of Immunology, School of Basic Medical Sciences, Tianjin Medical University, Tianjin, China; 3 Department of Biotechnology and Bioinformatics, Korea University 65692 , Sejong, Republic of Korea; 4 Interdisciplinary Graduate Program for Artificial Intelligence Smart Convergence Technology, Korea University 65692 , Sejong, Republic of Korea; Center for Microbial Dynamics & Infection, Atlanta, Georgia, USA

**Keywords:** *Pseudomonas aeruginosa*, virulence, quorum sensing, LasR, PhoB-PhoR

## Abstract

*Pseudomonas aeruginosa* is a major opportunistic pathogen that causes lung infections in patients with cystic fibrosis and chronic obstructive pulmonary disease. Loss-of-function mutations in the quorum-sensing regulatory gene *lasR* commonly arise during chronic infections, which are associated with exaggerated inflammation and accelerated decline in lung function. Here, in a murine cutaneous abscess model, infection with a Δ*lasR* mutant or a wild-type-Δ*lasR* mutant-mixed population resulted in higher bacterial loads and more severe tissue damage than infection by the wild-type strain. The Rhl and PQS quorum-sensing system genes, as well as phospholipase genes, were upregulated in the Δ*lasR* mutant and the wild-type-Δ*lasR* mutant-mixed population, which is mediated by PhoB in response to the *in vitro* and *in vivo* low-phosphate environments. We further demonstrate an auto-regulatory mechanism of PhoB and identify two LasR-regulated small RNAs that directly repress the translation of *phoB*. Overall, our results reveal a novel regulatory mechanism whereby mutation of *lasR* increases the pathogenesis of *P. aeruginosa*, providing an explanation for the more severe course of infection with the appearance of *lasR*-defective mutants.

**IMPORTANCE:**

*Pseudomonas aeruginosa* is an opportunistic pathogen that causes life-threatening infections. The bacterial quorum-sensing systems play important roles in coordinating gene expression during infection. Loss-of-function mutations in a quorum-sensing regulator gene *lasR* are commonly found in clinical isolates, which are associated with more rapid lung function decline. Here, in a murine cutaneous abscess model, we demonstrate that the presence of a *lasR*-defective mutant results in hyperproduction of virulence factors, increased antibiotic resistance, and more severe tissue damage, which resembles the human circumstance. We further identify the host environment signal and a novel regulatory pathway whereby mutation of *lasR* increases the bacterial pathogenesis. Our findings offer new insights into the LasR-mediated regulatory network in response to the host environment and provide clues to understand the lung disease progression driven by *lasR*-defective mutants.

## INTRODUCTION

During chronic infection, mutations in the pathogen population may give rise to cells with enhanced adaptation to the host environment and increased resistance to immune clearance and antibiotics (1–3). *Pseudomonas aeruginosa* is one of the leading pathogens that cause infections in patients with cystic fibrosis (CF) and chronic obstructive pulmonary disease (COPD) (4–7). The *lasR* gene encodes a quorum-sensing transcriptional regulator (8–10), and loss-of-function mutations in the *lasR* gene arise frequently in chronic infections, suggesting a strong selective pressure under *in vivo* conditions (11–15).

*P. aeruginosa* harbors three quorum-sensing systems, namely Las, Rhl, and PQS. Las and Rhl are acylhomoserine lactone-based systems. The Las system is composed of LasR and its cognate signal molecule N-(3-oxododecanoyl)-l-homoserine lactone (3-oxo-C12-HSL), which is synthesized by LasI. The Rhl system is composed of RhlR and its cognate signal molecule N-butyryl-l-homoserine lactone (C4-HSL), which is synthesized by RhlI (16). The signal molecules of the PQS system are 2-heptyl-3-hydroxy-1H-quinolin-4-one (PQS) and 2-heptyl-1H-quinolin-4-one (HHQ), produced by *pqsABCDE*, *phnAB,* and *pqsH* (17, 18). The signal molecules are recognized by a transcriptional regulator PqsR (also known as MvfR). The three quorum-sensing systems are interconnected, and the LasR system regulates both the Rhl and PQS systems (9, 19). Besides the core network, the quorum-sensing systems are also regulated by a variety of regulators and environmental signals, such as the alternative sigma factors RpoS (20, 21) and RpoN (22–25); the global regulators Vfr, GacA, QscR, and MvaT (26–29); the catabolite repression control protein Crc (30); the c-di-GMP signal molecule (31), etc. In addition, phosphate limitation has been shown to regulate the quorum-sensing systems through the PhoR-PhoB two-component regulatory system (32–35). The response regulator PhoB directly activates the expression of *lasI* by binding to a Pho box upstream of the coding region (32).

The *P. aeruginosa* quorum-sensing systems regulate more than 600 genes (approximately 12% of the genome), which comprise a large number of genes involved in the production of virulence factors, such as elastase, alkaline proteases, lectins PA-IL and PA-IIL, hydrogen cyanide, pyocyanin, rhamnolipids, *etc*. (36–40). Loss-of-function mutations in the *lasR* gene reduce the production of extracellular proteases (41). *In vitro* evolution assay in a synthetic CF sputum medium or using casein or bovine serum albumin as the sole carbon source demonstrated rapid emergence of *lasR* mutations (42, 43). The *lasR* mutants are frequently described as cheaters, as they can exploit public goods such as proteases produced by wild-type cells (44–46). Although LasR positively regulates the expression of multiple virulence genes, the rise of *lasR*-defective mutants during chronic lung infection is usually associated with increased inflammation and accelerated decline in lung function (47–50). Hennemann et al. demonstrated that infection with a *lasR*-deficient mutant resulted in a higher level of membranous intercellular adhesion molecule-1 (mICAM-1) in airway epithelial cells and subsequently more severe neutrophilic inflammation than that by the isogenic wild-type strain, which is due to the defective degradation of mICAM-1 by LasR-regulated extracellular proteases (48). Hoffman et al. demonstrated that a mutation in *lasR* renders the cell growth advantage and increased antibiotic resistance in CF airways, largely due to a metabolic shift (51). By using *in vitro* co-culture assays, Mould et al. demonstrated that citrate promotes RhlI expression in *lasR*-defective strains (52). However, it remains unclear how the *lasR* mutant subpopulation impacts the overall virulence factor production during infection.

In this study, we evaluated the effect of *lasR* mutation in bacterial pathogenesis by using a murine cutaneous abscess model, which was developed to study bacterial chronic infections (53). Compared with the wild-type strain alone, co-infection with the *lasR* mutant enhanced the pathogenesis. We found that the PhoB-mediated phosphate starvation response pathway is activated during infection, which activates the Rhl and PQS systems. Under phosphate limitation conditions, the *lasR* mutant exhibits stronger activation of the Rhl and PQS systems than the wild-type strain. We further identified two small RNAs through which LasR represses the *phoB* translation. Overall, our results characterized the expression of virulence genes in the *lasR* mutant during infection and elucidated the regulatory pathway, revealing a potential reason for the deterioration of lung functions with the emergence of such mutants during chronic human infections.

## RESULTS

### Co-infection with a *lasR*-deficient mutant enhances bacterial pathogenesis

**Fig 1:**
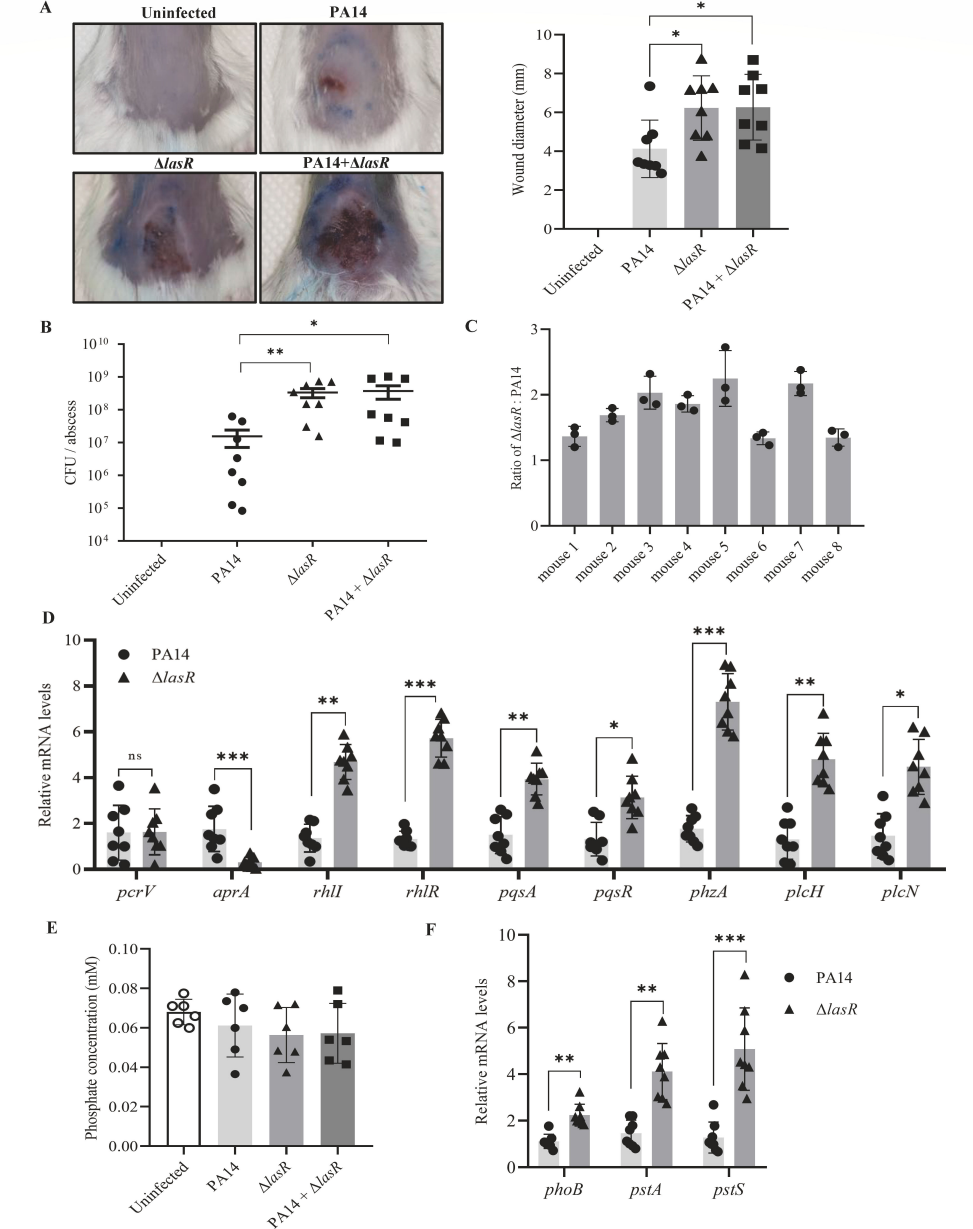
Co-infection with the Δ*lasR* mutant enhances bacterial pathogenesis. (**A**) Left: skin images of *P. aeruginosa* cutaneous infection on mice. A miniTn7 transposon carrying a gentamicin-resistant gene was inserted into the wild-type PA14. The resultant strain and an isogenic Δ*lasR* mutant, either alone or in combination (1:1), were injected (5 × 10^7^ CFU in 50 µL normal saline) under the thin skeletal muscle at the mouse dorsum; 50 µL of normal saline was injected into another group of mice as a negative control. The injection sites were marked with a blue marking pen. Photographs were taken 3 days post-injection. Right: quantification of wound sizes by a diameter of eight mice per group. (**B**) The CFU counts were determined 3 days post-infection. Bars represent medians, and error bars represent standard deviations. *, *P* < 0.05; **, *P* < 0.01 by ANOVA. (**C**) The ratios between the wild-type strain and the Δ*lasR* mutant in each of the eight co-infected mice. Bacteria isolated from the infected tissues were plated on LB plates with or without gentamicin. The CFU on the gentamicin plate was used to calculate the number of PA14. The Δ*lasR* mutant number was calculated as (CFU on the LB plate) − (CFUs on the gentamycin plate). (**D**) Relative mRNA levels of the indicated genes in PA14, the Δ*lasR* mutant, or the wild-type-Δ*lasR* combination from each individual infected mouse 3 days post-infection. *, *P* < 0.05; **, *P* < 0.01; ***, *P* < 0.001 by ANOVA. (**E**) The phosphate concentrations of the abscess pus of mice injected with the indicated strains or normal saline 2 days post-infection. (**F**) Relative mRNA levels of *phoB*, *pstS,* and *pstA* in PA14, the Δ*lasR* mutant, or the wild type-Δ*lasR* combination 3 days post-infection. **, *P* < 0.01; ***, *P* < 0.001 by ANOVA.

To evaluate the impact of the *lasR* loss-of-function mutation on bacterial virulence, we infected mice with wild-type PA14 and an isogenic Δ*lasR* mutant in a murine cutaneous abscess model. Infection with the Δ*lasR* mutant resulted in more severe tissue lesions and approximately 20-fold higher bacterial load than the wild-type strain (Fig. 1A and B), demonstrating enhanced virulence. We then examined whether there is an interplay between the two strains during infection. Mice were infected with wild-type PA14 and the Δ*lasR* mutant at a 1:1 ratio with the same total bacterial number as in the monostrain infection. Our assumption was that no bacterial interplay might result in intermediate bacterial load and tissue lesion. Infection with the combined strains resulted in similar bacterial loads and tissue lesions as the Δ*lasR* mutant (Fig. 1A and B). Meanwhile, the ratio between the Δ*lasR* mutant and the wild-type strain ranged from 1.2:1 to 2.7:1 3 days post-infection (Fig. 1C). These results indicate that the presence of the Δ*lasR* mutant enhances the pathogenesis of the bacterial population.

To understand the mechanism of the enhanced pathogenesis by the Δ*lasR* mutant, we examined the expression of virulence-related genes, including those involved in quorum sensing, the type III secretion system (T3SS), and tissue damage (pyocyanin and secreted proteases), using bacterial RNA isolated from each individual infected mouse. The expression level of the T3SS gene *pcrV* was not affected by the *lasR* mutation (Fig. 1D). However, the alkaline metalloproteinase gene *aprA* was downregulated in the Δ*lasR* mutant (Fig. 1D), presumably owing to the role of LasR in directly regulating its expression (54). Surprisingly, the expression levels of *rhlI*, *rhlR*, *pqsA*, *pqsR*, the pyocyanin synthesis gene *phzA,* and the phospholipase C genes *plcH* and *plcN* were higher in the Δ*lasR* mutant than those in the wild-type strain, indicating a negative regulatory role of the LasR on those genes *in vivo* (Fig. 1D).

### Mutation in *lasR* promotes the Rhl and PQS systems through the phosphate stress response pathway

We next investigated the *in vivo* signal and regulatory mechanism that upregulate the virulence genes in the Δ*lasR* mutant. Previous studies demonstrated that *plcH*, *plcN,* and the Rhl and PQS systems are positively regulated by the two-component regulatory system PhoBR in response to phosphate limitation (35, 55–57). We thus speculated that the PhoBR system was activated under an *in vivo* environment. Indeed, the expression of *phoB* and genes involved in phosphate uptake, namely, *pstS* and *pstA*, was higher in a low phosphate medium or *in vivo* than in a high phosphate medium (Fig. S1).

We then measured the phosphate concentrations in the infected tissues. In agreement with the upregulation of the phosphate stress response genes, the phosphate concentrations of the pus or subcutaneous tissue fluid (from uninfected mice) ranged from 0.035 to 0.08 mM (Fig. 1E), which is lower than the concentration (0.8 mM) reported to induce the PhoBR system (35, 58). Furthermore, the expression levels of *phoB*, *pstS,* and *pstA* were higher in the Δ*lasR* mutant or the wild-type-Δ*lasR* combination than in the wild-type strain (Fig. 1F), indicating a negative regulatory role of LasR on the PhoB pathway *in vivo*.

**Fig 2:**
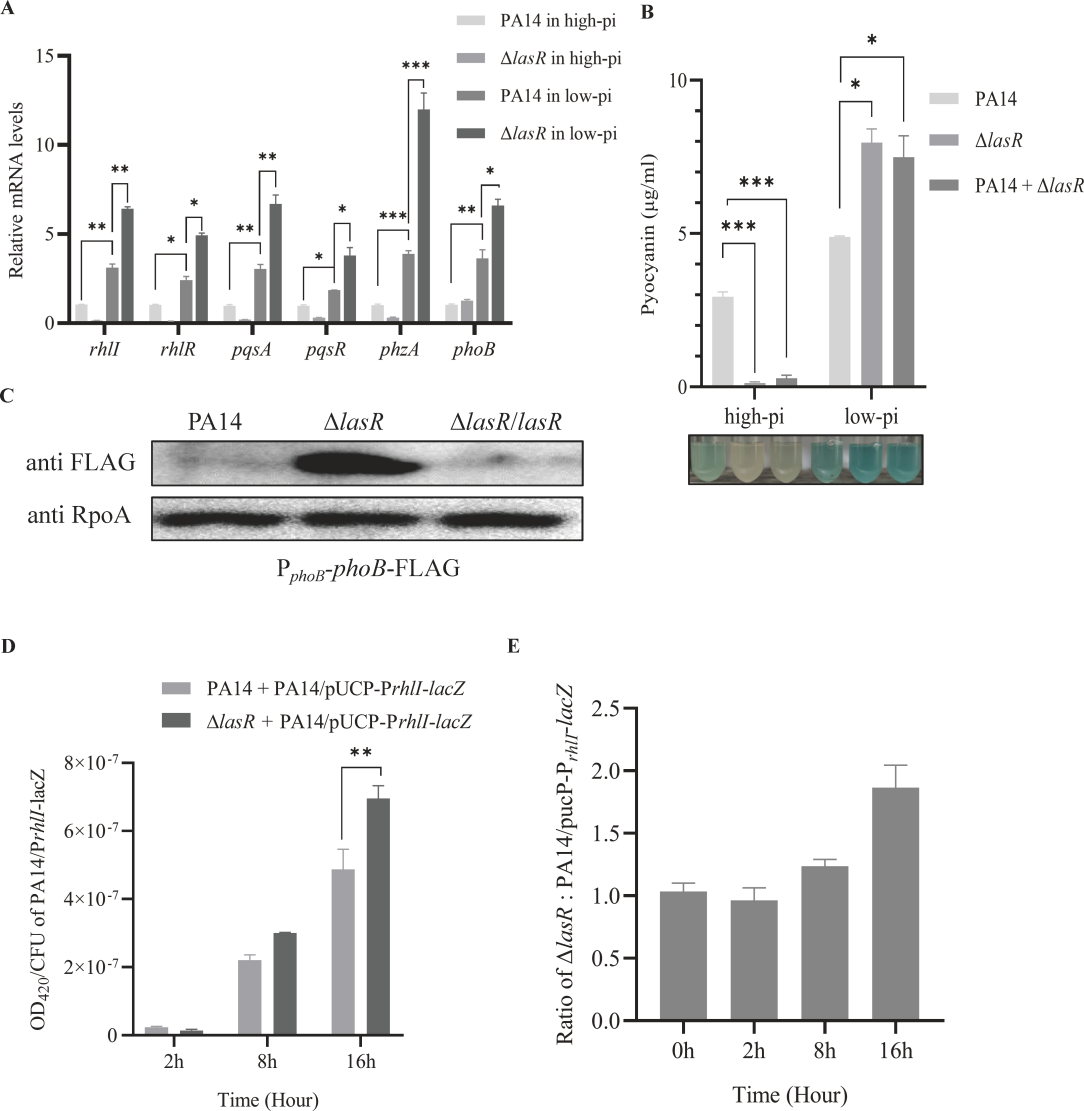
Mutation in *lasR* promotes the Rhl and PQS systems in response to phosphate limitation. (**A**) Relative mRNA levels of *rhlI*, *rhlR*, *pqsA*, *pqsR*, *phzA,* and *phoB* of PA14 and the Δ*lasR* mutant in high and low phosphate media. *, *P* < 0.05; **, *P* < 0.01; ***, *P* < 0.001 by ANOVA. (**B**) Pyocyanin concentrations of overnight cultures of PA14, the Δ*lasR* mutant, and the PA14-Δ*lasR* combination in high and low phosphate media. The final values have been normalized by the OD_600_ of each bacterial culture. Data represent the mean ± standard deviation of assays performed in triplicate and are representative of three independent experiments with similar results. *, *P* < 0.05; ***, *P* < 0.001 by ANOVA. (**C**) Bacteria containing the *phoB*-Flag driven by its native promoter were grown to an OD_600_ of 1.0 in the low phosphate medium. The levels of PhoB-FLAG and RpoA were determined by western blot. The same samples were run on two separate gels for the blotting of PhoB-Flag and RpoA, respectively. Data shown are representative of three independent experiments with similar results. PA14 or the Δ*lasR* mutant was co-cultured with PA14 containing the *rhlI* promoter *lacZ* transcriptional fusion (pUCP-P*_rhlI_-lacZ*) at a ratio of 1:1 in the low-phosphate medium. At indicated time points, β-galactosidase activities (**D**) and the ratio between PA14/pUCP-P*_rhlI_-lacZ* and the Δ*lasR* mutant (**E**) were determined.

Under *in vitro* high phosphate conditions, mutation of *lasR* reduced the expression levels of *rhlI*, *rhlR*, *pqsA*, *pqsR*, *phzA,* and pyocyanin production without affecting the expression of *phoB*, demonstrating a positive role of LasR in regulating the Rhl and PQS systems (Fig. 2A and B). However, the low phosphate condition increased the expression of not only *phoB* but also the quorum-sensing genes as well as pyocyanin production in both wild-type PA14 and the Δ*lasR* mutants, with a greater extent in the Δ*lasR* mutant (Fig. 2A and B).

We further verified the expression of PhoB by using a FLAG-tagged *phoB* gene driven by its native promoter (P*_phoB_-phoB*-FLAG). Mutation of *lasR* increased the expression of the PhoB-FLAG in the low phosphate medium (Fig. 2C). Previously, we demonstrated that PhoB-mediated hyperproduction of pyocyanin increases bacterial resistance to β-lactam antibiotics (59). In the low phosphate medium, the Δ*lasR* or the wild-type-Δ*lasR* combination population displayed higher survival than the wild-type alone following ceftazidime treatment (Fig. S2). Furthermore, following growth with the wild-type strain for 16 h, the Δ*lasR* mutant population rose from 50% to 65% in the low phosphate medium, whereas in the high phosphate medium, the Δ*lasR* mutant took over the whole population (Fig. S3).

We then examined whether the presence of the Δ*lasR* mutant can enhance the quorum-sensing system in the wild-type strain under phosphate limitation. Wild-type PA14 containing an *rhlI* promoter-*lacZ* transcriptional fusion (P*_rhlI_-lacZ*) was co-cultured with the wild-type PA14 or the Δ*lasR* mutant in the low phosphate medium. After 16 h, co-culture with the Δ*lasR* mutant resulted in higher LacZ activity compared with that in the PA14(P*_rhlI_-lacZ*)-PA14 population (Fig. 2D and E), indicating an enhancement of the *rhl* gene expression in the wild-type strain by the Δ*lasR* mutant. Collectively, these results indicate that loss of *lasR* enhances the expression of *phoB* and the Rhl and PQS system genes under the phosphate limitation condition.

**Fig 3:**
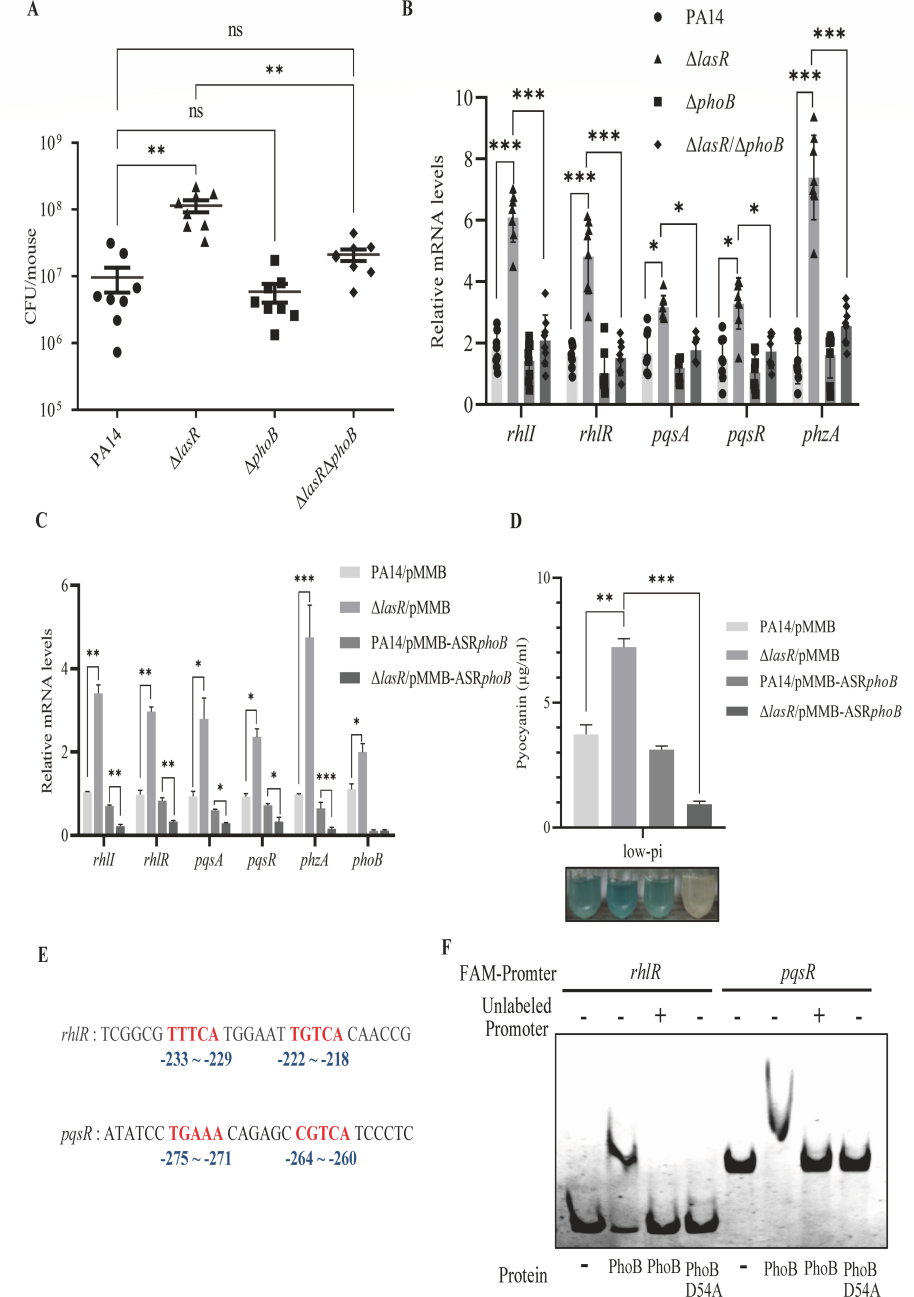
Mutation in *lasR* enhances the Rhl and PQS systems through PhoB. (**A**) Relative mRNA levels of *rhlI*, *rhlR*, *pqsA*, *pqsR,* and *phzA* of PA14, Δ*lasR*, Δ*phoB,* and the Δ*lasR*Δ*phoB* double mutant in the low phosphate medium. *, *P* < 0.05; ***, *P* < 0.001 by ANOVA. (**B**) Bacterial colonization in the cutaneous abscess model infected by PA14, Δ*lasR*, Δ*phoB,* and the Δ*lasR*Δ*phoB* double mutant. CFU counts were determined 3 days post-infection. Bars represent medians, and error bars represent standard deviations. **, *P* < 0.01 by ANOVA. (**C**) Relative mRNA levels of *rhlI*, *rhlR*, *pqsA*, *pqsR, phzA,* and *phoB* of PA14, the Δ*lasR* mutant with or without expressing the *phoB* antisense RNA in the low phosphate medium. *, *P* < 0.05; **, *P* < 0.01; ***, *P* < 0.001 by ANOVA. (**D**) Pyocyanin concentrations of overnight cultures of PA14, the Δ*lasR* mutant with or without expressing the *phoB* antisense RNA in the low phosphate medium. The final values were normalized by the OD_600_ of each bacterial culture. Data represent the mean ± standard deviation of assays performed in triplicate and are representative of three independent experiments with similar results. *, *P* < 0.05; ***, *P* < 0.001 by ANOVA. (**E**) Potential Pho boxes in the promoter regions of *rhlR* and *pqsR* are shown in red. The numbers indicate the distance upstream of the start codons. (**F**) Binding of PhoB and PhoB D54A to the promoter regions of the indicated genes was examined by competitive EMSA. Fifty nanograms FAM-labeled *rhlR* or *pqsR* promoter region DNA (FAM-promoter) was incubated alone, with 0.1 µM purified PhoB protein, PhoB protein plus 50-fold excess of the corresponding unlabeled DNA fragments (unlabeled-promoter), or 0.1 µM purified PhoB D54A protein.

We next examined the role of PhoB in regulating the virulence and quorum-sensing systems by assaying a Δ*phoB* and a Δ*lasR*Δ*phoB* in the animal infection model. Deletion of the *phoB* gene in the Δ*lasR* mutant reduced the bacterial load and expression of the quorum-sensing system genes (Fig. 3A and B), demonstrating a role of PhoB in activating the quorum-sensing systems under *in vivo* conditions. To further verify the regulatory roles of LasR and PhoB, we attempted to grow the bacteria in a low-phosphate medium. However, deletion of *phoB* severely impaired the bacterial growth under this condition (Fig. S4). We thus suppressed the expression of *phoB* by overexpressing an antisense RNA (60). As shown in Fig. 3C and D, suppression of PhoB in the Δ*lasR* mutant reduced the expression of the quorum-sensing genes and pyocyanin production under the low phosphate growth condition.

To verify that PhoB directly activates the Rhl and PQS systems in response to phosphate limitation, we performed an electrophoretic mobility shift assay (EMSA) using a DNA fragment upstream of the promoter regions of *rhlR* and *pqsR*, which contain the Pho box sequences (Fig. 3E). Since phosphorylation of the PhoB D54 residue is required for the dimerization and binding of PhoB to target DNA, we used a PhoB D54A mutant as a negative control (32, 61, 62). As shown in Fig. 3F, PhoB bound to the DNA fragments, which was abolished by the D54A mutation (58). In combination, these results demonstrate that mutation of *lasR* results in upregulation of *phoB* under *in vivo* and *in vitro* phosphate limitation environments, which subsequently enhances the Rhl and PQS systems.

### LasR represses the translation of *phoB* through two small RNAs

**Fig 4:**
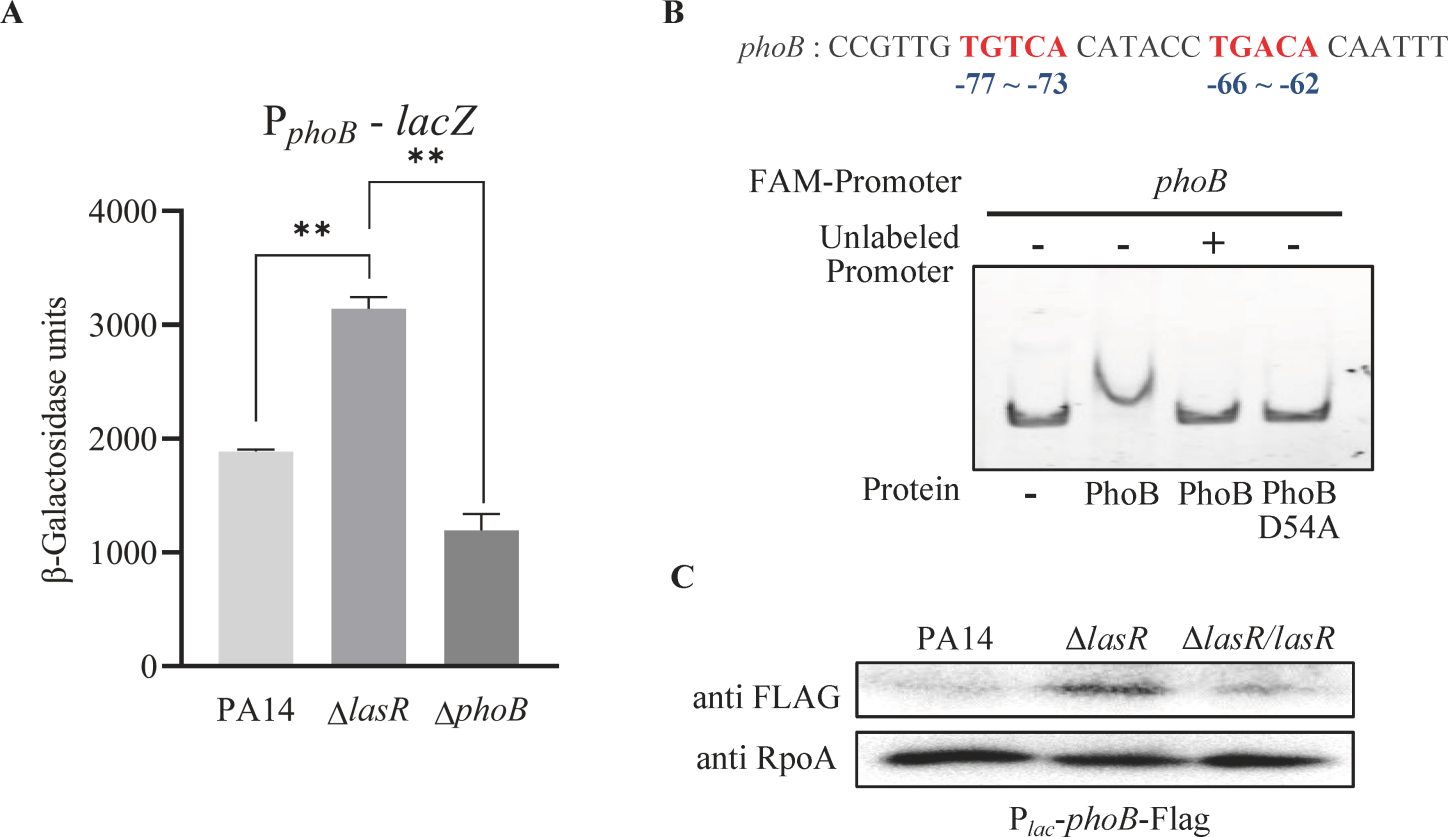
Mutation of *lasR* increases the *phoB* expression. (**A**) The wild-type PA14, Δ*lasR,* and Δ*phoB* carrying the P*phoB-lacZ* transcriptional fusion were grown in LB to an OD_600_ of 1.0, followed by β-galactosidase assays. **, *P* < 0.01 by ANOVA. (**B**) Upper panel, the predicted Pho box in the *phoB* promoter region is shown in red; lower panel, competitive EMSA analysis of PhoB and PhoB D54A binding to the promoter regions of *phoB*. Fifty nanograms FAM-labeled *phoB* promoter region DNA (FAM-promoter) was incubated alone, with 0.1 µM purified PhoB protein, PhoB protein plus 50-fold excess of the corresponding unlabeled DNA fragments (unlabeled-promoter), or 0.1 µM purified PhoB D54A protein. (**C**) Bacteria containing the *phoB*-Flag driven by a *lac* promoter (P*lac-phoB*-Flag) were grown to an OD_600_ of 1.0 in LB. The levels of PhoB-FLAG and RpoA were determined by western blot. Data shown are representative of three independent experiments with similar results.

We next explored the mechanism by which LasR represses *phoB* expression. By using a P*_phoB_-lacZ* transcriptional fusion, we found that mutation of *lasR* increased the promoter activity of *phoB* in LB (Fig. 4A), as well as in the high and low phosphate media (Fig. S5). A previous ChIP-Seq assay revealed a Pho box in the promoter region of *phoB* (58) (Fig. 4B; Fig. S6), indicating an autoregulatory mechanism. Indeed, PhoB binds to its own promoter region, which requires the phosphorylatable Asp54 (Fig. 4B; Fig. S6). In addition, deletion of *phoB* reduced the expression of P*_phoB_-lacZ* (Fig. 4A). These results demonstrate an autoactivation of *phoB*.

**Fig 5:**
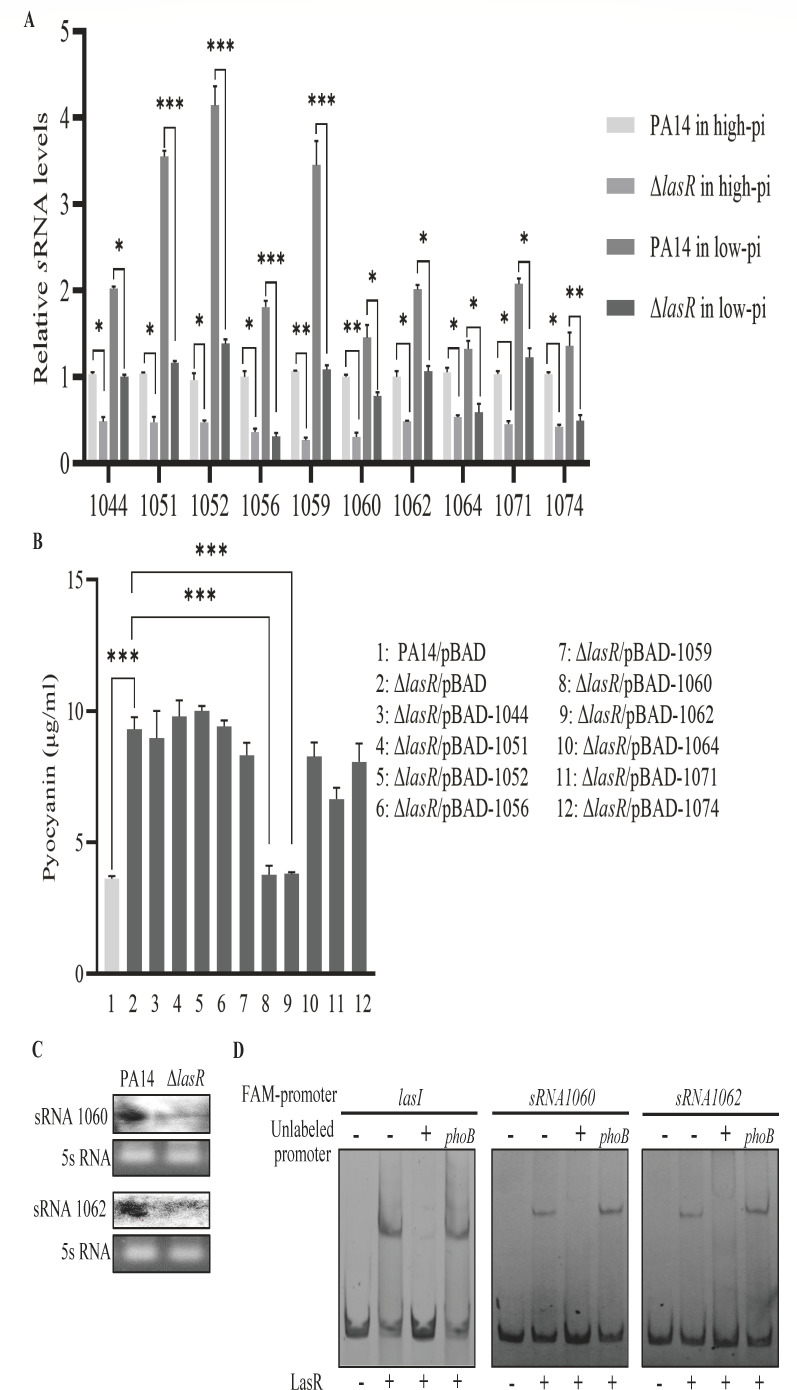
LasR represses the translation of *phoB* through two small RNAs. (**A**) Relative levels of the indicated sRNAs in PA14 and the Δ*lasR* mutant in the high and low phosphate media. *, *P* < 0.05; **, *P* < 0.01; ***, *P* < 0.001 by ANOVA. (**B**) Each sRNA was overexpressed in the Δ*lasR* mutant, followed by the determination of the extracellular pyocyanin concentrations after overnight growth. ***, *P* < 0.001 by ANOVA. (**C**) sRNA 1060 and sRNA 1062 were identified by northern blot in PA14 and the Δ*lasR* mutant. (**D**) The binding of the LasR protein to the promoter regions of sRNA 1060 and sRNA 1062 was examined by competitive EMSA. Fifty nanograms FAM-labeled *lasI*, sRNA 1060 or sRNA 1062 promoter region DNA (FAM-promoter) was incubated alone, with 1 µM purified LasR protein, LasR protein plus 50-fold excess of the corresponding unlabeled DNA fragments (unlabeled-promoter) or the unlabeled *phoB* promoter region as a negative competitor control.

To dissect whether LasR represses the transcription or translation of *phoB*, we constructed a C-terminal Flag-tagged *phoB* fusion driven by an exogenous *lac* promoter (P*_lac_-phoB*-Flag). Mutation of *lasR* increased the expression of the PhoB-FLAG fusion (Fig. 4C), demonstrating a translational repression by LasR. To elucidate the regulatory mechanism, we performed transcriptomic analysis on wild-type PA14 and the Δ*lasR* mutant; 18 small RNAs (sRNAs) were downregulated in the Δ*lasR* mutant (Table S1), of which 10 were predicted to target the *phoB* mRNA as analyzed by IntaRNA. qRT-PCR verified the downregulation of the 10 sRNAs in the Δ*lasR* mutant in both the high and low phosphate media (Fig. 5A). We then overexpressed each of the sRNAs in the Δ*lasR* mutant and found that sRNA1060 and sRNA1062 reduced pyocyanin production in the low phosphate medium (Fig. 5B).

We thus focused on the regulation and functional mechanisms of the sRNA1060 and sRNA1062. Northern blot verified the downregulation of the sRNAs in the Δ*lasR* mutant (Fig. 5C). In addition, EMSA results demonstrated the binding of LasR to the promoter regions of sRNA1060 and sRNA1062 (Fig. 5D; Fig. S7). Collectively, these results demonstrate that LasR directly regulates the sRNAs sRNA1060 and sRNA1062.

**Fig 6:**
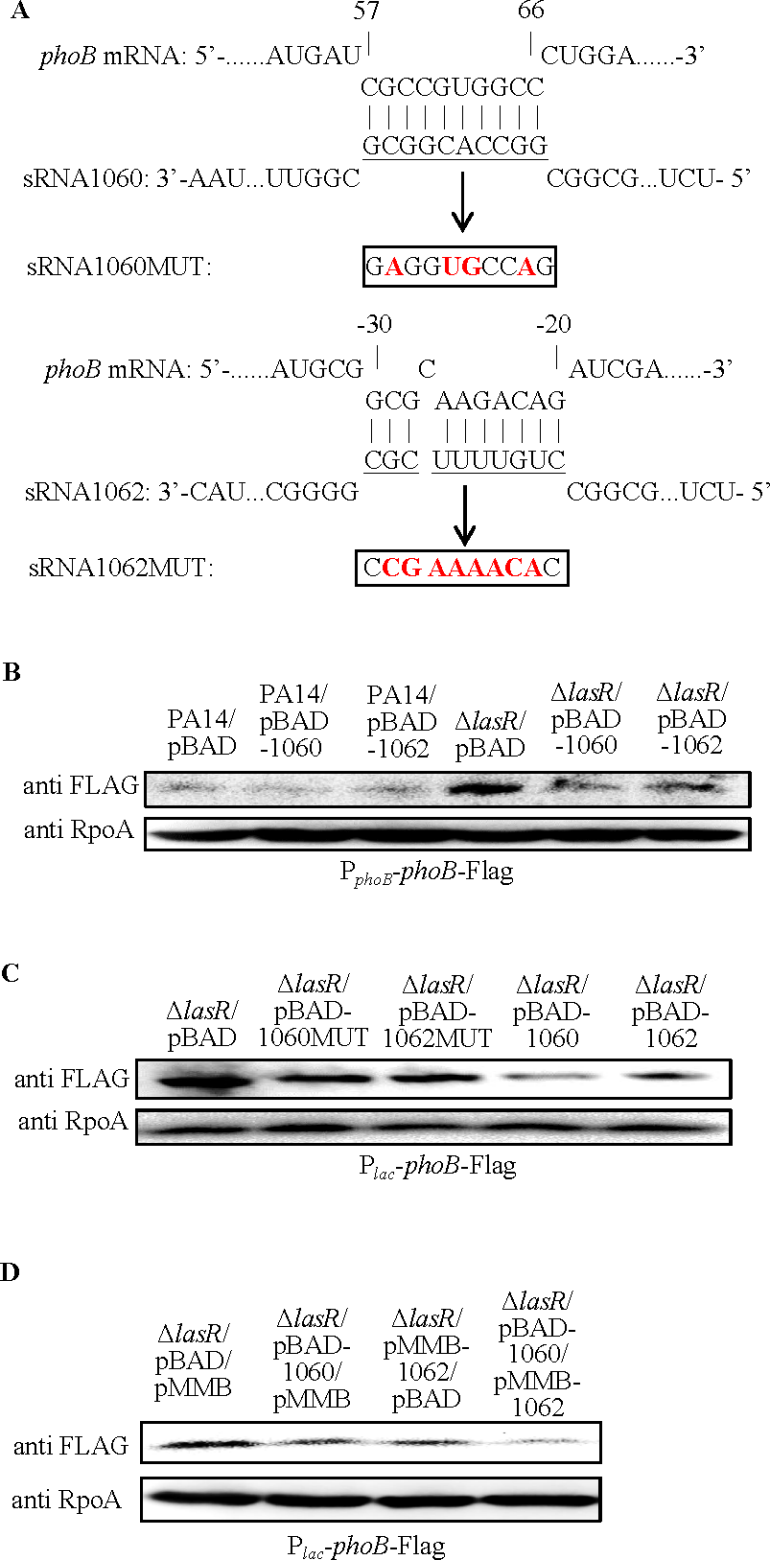
sRNA1060 and sRNA1062 repress PhoB translation. (**A**) Potential interactions between sRNA1060, sRNA1062, and the *phoB* mRNA were predicted by intaRNA. The underlined nucleotides in sRNA1060 and sRNA1062 were replaced by the boxed sequences, resulting in sRNA1060MUT and sRNA1062MUT, respectively. (**B**) sRNA1060 or sRNA1062 was overexpressed in PA14 and the Δ*lasR* mutant. Bacteria containing the *phoB*-Flag driven by its native promoter (P*phoB-phoB*-Flag) were grown to an OD_600_ of 1.0 in LB. The levels of PhoB-FLAG and RpoA were determined by western blot. Data shown are representative of three independent experiments with similar results. (**C**) sRNA1060, sRNA1062, or their mutants were overexpressed in the Δ*lasR* mutant. Bacteria containing the P*lac-phoB*-Flag were grown to an OD_600_ of 1.0 in LB. The levels of PhoB-FLAG and RpoA were determined by western blot. Data shown are representative of three independent experiments with similar results.

sRNA1060 and sRNA1062 were predicted to bind to the coding sequence and 5′-UTR of the *phoB* mRNA, respectively (Fig. 6A). Overexpression of sRNA1060 and sRNA1062 in the Δ*lasR* mutant reduced the expression of the PhoB-FLAG from both the P*_phoB_-phoB*-Flag and P*_lac_-phoB*-Flag (Fig. 6B and C). Additionally, co-overexpression of sRNA1060 and sRNA1062 further reduced PhoB expression (Fig. 6D). Mutation of the predicted binding sequences of sRNA1060 and sRNA1062 on the *phoB* mRNA attenuated the translational repression (Fig. 6A and C).

Searching through 7,906 available *P. aeruginosa* genomes in the *Pseudomonas* database (https://www.pseudomonas.com) (63) revealed the presence of sRNA1060 and sRNA1062 homologs in 50.6% and 16.4% of the strains, respectively (Table S2). Although only 0.6% isolates contain both of the sRNAs, 66.4% of all the isolates contain at least one of the two sRNAs (Fig. S8). The widely used reference strain PAO1 harbors sRNA1060 but not sRNA1062. Deletion of *lasR* in PAO1 resulted in downregulation of sRNA1060, upregulation of the Rhl, PQS, and pyocyanin synthesis genes, as well as increased pyocyanin production under the low phosphate condition (Fig. S9A through C). In addition, overexpression of either sRNA1060 or sRNA1062 in PAO1 decreased the expression of PhoB (Fig. S9D). These results indicate a conserved regulatory mechanism in PAO1.

## DISCUSSION

*P. aeruginosa lasR*-defective mutants are commonly identified in clinical niches like the lungs of CF patients (14, 64). Despite being recognized as a “social cheater,” the appearance of *lasR* mutants in CF lungs is associated with more severe inflammation and accelerated lung function decline (47, 65). It has been demonstrated that loss of *lasR* increases neutrophil recruitment and accumulation of proinflammatory cytokines due to the loss of bacterial protease-dependent degradation of cell surface proteins and cytokines (48, 65). Meanwhile, the Rhl system remains active in many *lasR*-defective isolates from CF patients (14, 66). Oshri et al. and Kostylev et al. found that mutations in the *mexT* gene are further evolved from the *lasR* mutant, which leads to activation of the Rhl system (67, 68). Mould et al. demonstrated that citrate cross-feeding from *lasR*^+^ to *lasR*^-^ strains activates the Rhl system in the *lasR*^-^ strain (52). By using an *ex vivo* pig lung (EVPL) model, Harrison et al. demonstrated that *lasR* mutants are more adapted to the infection environment (69). Here, we demonstrated that under the phosphate limitation condition, the phosphorylated PhoB protein directly upregulates the expression of *rhlR* and *pqsR* by binding to their promoter regions, leading to the activation of the Rhl and PQS systems. Phosphate limitation has been shown to induce the production of virulence factors in *P. aeruginosa* (33, 56, 70–74). Jensen et al. identified Pho box sequences in the *rhlR* and *pqsR* promoter regions, indicating a role of PhoB in regulating their expression (35). By using a mouse surgical hepatectomy model, Long et al. demonstrated that the surgical injury leads to the depletion of intestinal phosphate, which activates the virulence of *P. aeruginosa* and causes lethal gut-derived sepsis (70). By using a murine cutaneous abscess model, we found that the *P. aeruginosa* phosphate transportation system was activated *in vivo,* and the phosphate concentrations in the infected tissues were around 0.035–0.08 mM, which is much lower than those used in *in vitro* assays to activate the PhoR-PhoB system (35, 58). Clinical studies revealed that hypophosphatemia is associated with increased risk of septic shock and mortality in patients with *P. aeruginosa* infections (75, 76). More importantly, transcriptomic analysis demonstrated upregulation of the phosphate transportation genes (e.g., *pstA*, *pstS*) in *P. aeruginosa* in sputum from individuals with cystic fibrosis compared with the counterparts grown *in vitro* (77, 78), indicating a phosphate limitation environment *in vivo*.

**Fig 7:**
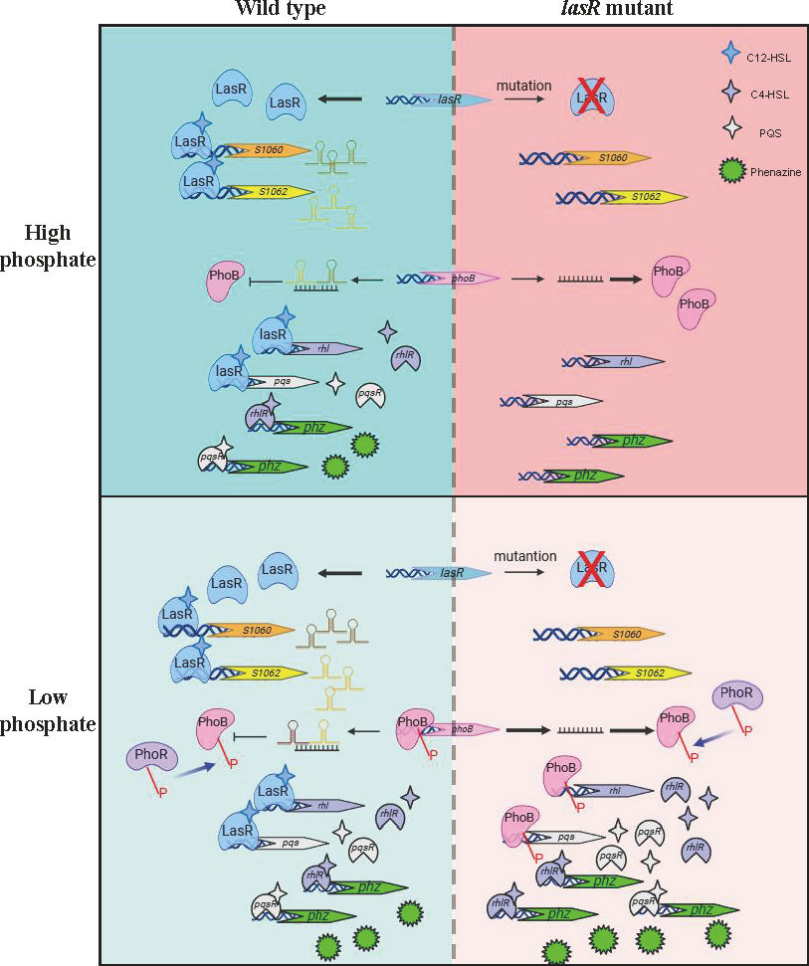
Model illustrating the mechanism of the *lasR* mutation caused hyper-activation of Rhl and PQS systems in a phosphate limitation environment. Top, in a high phosphate condition, mutation of *lasR* leads to downregulation of the Rhl and PQS QS systems. Although the repression of PhoB is removed by downregulation of sRNA1060 and sRNA1062, PhoB is unable to activate the Rhl and PQS systems due to the lack of phosphorylation. Bottom, in a low phosphate condition, the downregulation of sRNA1060 and sRNA1062 in the *lasR*-defective mutant results in derepression of the translation of PhoB. In response to the phosphate limitation condition, PhoB is phosphorylated by PhoR, which increases the transcription of *phoB*, *rhlR,* and *pqsR* by directly binding to their promoter regions, thus activating the downstream QS system genes.

Conaway et al. recently reported upregulation of *phoB* and PhoB-regulated genes in a Δ*lasR* mutant at 0.7 mM phosphate (79). In a low phosphate medium, we observed hyper-activation of the Rhl and PQS systems and hyper-production of pyocyanin in both a *lasI* mutant and a *lasR* mutant. Through transcriptomic analysis, we identified two novel small RNAs, sRNA1060 and sRNA1062, that are positively regulated by LasR. sRNA1060 and sRNA1062 repress the translation of *phoB* by directly targeting its coding region and 5′-UTR, respectively. The regulatory pathway is summarized in Fig. 7.

Previously, we found that a mutation in a constitutive phosphate transporter gene *pitA* increased bacterial resistance to β-lactam antibiotics by hyper-production of pyocyanin (59). We later demonstrated that a mutation in *pitA* reduces intracellular phosphate concentration, which activates the PhoRB system and subsequently upregulates the pyocyanin biosynthesis genes and increases bacterial resistance to β-lactam antibiotics (34). In this study, we found that loss of *lasR* increased bacterial survival following ceftazidime treatment. More importantly, the mixed population of wild-type and the *lasR* mutant displayed the highest survival following the treatment (Fig. S2), which might impair the treatment efficacy. In addition, infection of mice with the mixed population (WT:Δ*lasR* = 1:1) resulted in more severe tissue damage and higher bacterial load than the wild-type strain alone. Three days following the infection, the ratios of the wild-type and Δ*lasR* strains ranged between 1:1.2 and 1:2.7. Under *in vitro* low phosphate conditions, the ratio between the wild-type and Δ*lasR* strains remains relatively stable (from 1:1 to 1:2) during 16 h, whereas under high phosphate conditions, the *lasR* mutant gradually takes over the population (Fig. S3) (80). These results indicate that under low phosphate conditions, the presence of the *lasR* mutant might produce more public goods, which promote the virulence and antibiotic resistance of the mixed population.

Previously, Rumbaugh et al. found that the *lasR* mutant was attenuated in a burn wound infection model (81, 82). The *lasR* mutant is defective in producing the elastases LasA, LasB, exotoxin A, and alkaline proteases AprA, which might play more important roles in bacterial pathogenesis in the burn wound model (82). However, *lasR* mutations have been shown as an adaptive evolution in chronic wounds and lung infections (14, 64, 83). These results indicate that the various nutrient conditions in different infected tissues might impact the bacterial physiology and regulatory pathways (such as the PhoBR system) differently, leading to diverse evolution trajectories. Further studies are warranted to investigate the nutritional conditions of different infection sites and their effects on bacterial gene expression and pathogenesis.

By searching through the *P. aeruginosa* genomes in the Pseudomonas database (https://www.pseudomonas.com) (62), we found that 66.4% of the isolates contain at least one of the two sRNAs (Fig. S8). Among the isolates carrying sRNA1060, we randomly chose 10 isolates from cystic fibrosis patients or chronic wounds each. sRNA1062 is present in three and two isolates from CF patients and chronic wounds, respectively. A thorough analysis of the sequences and strain information will provide insight into the conservation of the regulatory mechanism.

The QS systems of *P. aeruginosa* exhibit remarkable diversity, and different QS systems can collaborate temporally or spatially (84–86). The “Las system-sRNAs-PhoB-Rhl and PQS systems” pathway we discovered here reveals a new regulatory mechanism in response to phosphate limitation. This pattern may also serve as an example demonstrating how bacteria switch between cheater and cooperator under different environments. O'Connor et al*.* comprehensively analyzed QS gene mutations in *P. aeruginosa* strains from various environments (13). They found the high frequency of loss-of-function *lasR* mutations across all tested environments, including cystic fibrosis lungs, wounds, and non-infection environmental sources. Besides, the PQS system genes *pqsA*, *pqsL*, *pqsH,* and QS-related *mexT* genes are highly variable. However, the *rsaL* gene that encodes a negative regulator of the Las system is conserved (13). These results demonstrated that QS mutations are common in *P. aeruginosa*, which sheds light on the evolutionary dynamics of QS systems and their roles in infection and environmental adaptation. We found that the sRNAs sRNA1060 and sRNA1062 are present in approximately 60% of *P. aeruginosa* strains. This regulatory mechanism may depend on the genetic background of the strain (particularly the composition and status of its QS systems) as well as the specific environment it is in (such as the chronic wound microenvironment versus the CF lung microenvironment). It will be interesting to investigate the other 40% strains, whether mutation of *lasR* affects the PhoBR system under phosphate limitation conditions, or other regulatory mechanisms are involved.

Overall, this study provides an explanation for the deteriorated lung functions caused by *lasR*-deficient mutant strains in the clinic. Therefore, it is likely that the *lasR* mutants produce a larger number of autoinducers of the Rhl and PQS systems, extracellular proteases, and virulence factors during infection, which might enhance the pathogenesis of the bacterial population.

## MATERIALS AND METHODS

### Bacterial strains, plasmids, and culture media

The bacterial strains, plasmids, and primers used in this study are listed in Table S3. Bacterial cells were cultured in Luria–Bertani (LB) medium (5 g/L NaCl, 5 g/L yeast extract, and 10 g/L tryptone) at 37°C with shaking (200 rpm). To prepare plates, 1.5% (wt/vol) agar was added. The phosphate control medium is composed of 20 g of pancreatic digest of gelatin (Difco), 0.14 g MgCl_2_ (Sigma), and 10 g K_2_SO_4_ (Sigma) per liter of H_2_O. 4 mM and 0.2 mM of KH_2_PO_4_ were added for the high and low phosphate media, respectively.

### Animal experiment

The murine cutaneous abscess model was performed as previously described with minor modifications (53). In brief, 1 day before injection, the fur on the back of each of 6- to 8-week-old female BALB/c mice was removed by shaving and application of chemical depilatories. Bacteria were grown to an OD_600_ of 1.0 in LB medium at 37°C. Prior to injection, bacterial cells were washed twice with sterile saline solution and resuspended to a concentration of 1 × 10^8^ CFU/mL in sterile saline solution; 50 µL bacterial solution or sterile saline control was injected subcutaneously into the center of the glabrous part of the back. At 3 days post-infection (hpi), the mice were sacrificed by CO_2_. Skin abscesses and accumulated pus were excised and homogenized in sterile saline solution. Bacterial loads were enumerated by serial dilution and plating. For the co-infection experiments, the miniTn7 transposon containing a gentamicin-resistant gene was inserted into the wild-type PA14 (87). Bacteria isolated from the infected tissue were plated on LB plates with or without 50 µg/mL gentamycin. Colony numbers on the gentamycin plates represent the amount of the wild-type PA14. The number of the Δ*lasR* mutant was calculated by subtracting the CFU on the gentamycin plate from the CFU on the antibiotic-free LB plate. The experiments were performed at least twice independently with six animals per group.

### RNA isolation and quantitative real-time PCR

For samples from *in vivo* assays, bacteria in the homogenized skin abscesses and pus were collected by centrifugation and washed with saline solution twice. Total bacterial RNA was isolated by using the Trizol Reagent (Thermo Fisher Scientific, USA). For *in vitro* assays, bacteria were grown to the indicated growth phases in high or low phosphate medium. Total bacterial RNA was extracted by using the RNAprep pure cell/bacteria kit (Tiangen Biotec, Beijing, China). cDNA was synthesized with random primers and a reverse transcriptase (TAKARA, Dalian, China). Quantitative real-time PCR (qRT-PCR) was performed by using a CFX Connect Real-Time system (Bio-Rad, USA). The indicated specific primers, cDNA and a SYBR II green supermix (Bio-Rad, Beijing, China), were mixed in a total volume of 20 µL. The 30S ribosomal protein gene *rpsL* was used as the internal control. Data shown were from three biological replicates and represent the mean ± standard deviation.

### RNA sequencing

The wild-type strain and the Δ*lasR* mutant were cultured in high or low phosphate medium at 37°C to the log phase (OD_600_ = 1.0). Total bacterial RNA was isolated by using an RNAprep PureCell/Bacteria Kit (Tiangen Biotec, Beijing, China). RNA sequencing was performed by Azenta Life Sciences (Suzhou, China) as previously described (88). Briefly, after quantifying and qualifying, 1 µg RNA of each sample was used for library preparation. After depleting rRNA by an Ribo-Zero rRNA Removal Kit (Illumina, San Diego, CA, USA), the remaining RNA was reverse-transcribed with random primers and ProtoScript II Reverse Transcriptase, followed by synthesis of the complementary strand with Second-strand Synthesis Mix (Illumina, San Diego, CA, USA). The double-stranded cDNA was purified by beads and then treated with End Prep Enzyme Mix to repair both ends and add a dA-tailing in one reaction, followed by a T-A ligation to add adaptors to both ends. The adaptor-ligated 300-400 bp DNA fragments were enriched using beads. Each DNA sample was then amplified by PCR using Illumina PCR Primer Cocktail. Sequencing, image analysis, and base calling were carried out by the HiSeq, NovaSeq, and MGI2000 instruments. The RNA-seq raw data have been deposited in the NCBI (PRJNA1070051).

### Phosphate concentration determination

The inorganic phosphate concentration was determined as previously described by using a Fluorimetric Phosphate Assay Kit (AAT Bioquest, USA) (60). To determine the phosphate concentration of pus isolated from abscesses of mice, 40 µL of the 10-fold dilution (by deionized water) of the pus solution was mixed with 40 µL of the assay buffer, and 20 µL of the phosphate sensor working solution (provided by the kit) in each well of a 96-well plate. After incubation at room temperature for 45 min in the dark, the fluorescence was monitored by a fluorometer (Varioskan Flash; Thermo Scientific) at the excitation/emission of 540/590 nm. The phosphate concentrations were calculated by a standard curve (*Y* = 2.993*X* − 4.0117). Data represent the mean ± standard deviation of the results from three biological replicates.

### β-Galactosidase assay

The β**-**galactosidase assay was performed as previously described with modifications (88). Overnight bacterial cultures were diluted 1:100 into the indicated media. After growing to an OD_600_ of 1.0, 0.5 mL bacteria were collected by centrifugation and resuspended in 1.5 mL Z-buffer (60 mM Na_2_HPO_4_, 40 mM NaH_2_PO_4_, 50 mM β-mercaptoethanol, 10 mM KCl, and 1 mM MgSO_4_); 1 mL of the solution was used to measure OD_600_, and 0.5 mL of the solution was transferred to a new tube and then added with 10 µL chloroform and 10 µL 0.1% SDS. After vortexing for 10 s, 0.1 mL orthonitrophenyl-galactopyranoside (ONPG; 4 mg/mL) (BBI Life Sciences) was added to the mixture and incubated at 37°C. When the mixture turned light yellow, 0.5 mL 1 M Na_2_CO_3_ was added immediately, and the reaction time was recorded. The OD_420_ was measured after centrifugation at 16,000 × *g* for 5 min, and the β**-**galactosidase activity (Miller units) was calculated as 1,000 × OD_420_/*T*_min_/*V*_mL_/OD_600._ Data represent the mean ± standard deviation of the results from three biological replicates.

### Pyocyanin quantification

The overnight bacterial culture was centrifuged at 12,000 × *g* for 1 min; 1 mL supernatant was transferred to a new tube and mixed with 0.6 mL chloroform. After vortexing and centrifuging, the chloroform layer was collected and mixed with 0.2 mL of 0.2 N HCl. The upper layer was collected to measure the absorbance at 520 nm. The concentration of pyocyanin (μg/mL) was based on multiplying the absorbance value by 17.072. Data represent the mean ± standard deviation of the results from three biological replicates.

### Ceftazidime time-kill assays

Antibiotic time-kill assays were performed as described previously (89). Overnight cultures of bacteria were grown to an OD_600_ of 1.0 in the low phosphate medium. Each of the indicated strains was adjusted to 10^8^ CFU/mL in 3 mL fresh low phosphate medium and subjected to 8 µg/mL ceftazidime treatment at 37°C with shaking (200 rpm). The bacterial survival was determined by plating at the indicated time points. Data represent the mean ± standard deviation of the results from three biological replicates.

### Western blot

Equal amounts of bacteria were collected by centrifugation, the pellet was mixed with loading buffer, and boiled at 99°C for 15 min. The samples were loaded on the 12% sodium dodecyl sulfate-polyacrylamide (SDS-PAGE) gels for electrophoresis. Separated proteins were transferred onto the PVDF membranes (Millipore, USA). Due to the close molecular weights of the PhoB-Flag and RpoA, the same protein samples were run on two gels and blotted on two membranes. After blocking with 5% milk, the membrane was incubated with rabbit monoclonal anti-Flag antibody (Sigma, USA) or a mouse monoclonal anti-RpoA antibody (CST, USA) overnight at 4°C. After washing, membranes were incubated with the corresponding HRP-conjugated antibody anti-rabbit IgG (Promega) or anti-mouse IgG (Promega). The membrane was washed with PBST four times. Signals were detected by an ECL Plus kit (Millipore) and visualized using a Bio-Rad molecular imager (ChemiDocXRS).

### Electrophoretic mobility shift assay

EMSA assays were performed as described previously with minor modifications (90). The DNA probe was amplified by PCR using the primers in Table S3. The proteins were purified through the nickel columns. Indicated DNA fragments (200 ng) were incubated with purified protein in a 20 µL reaction buffer (4% glycerol, 10 mM pH 7.6 Tris-HCl, 5 mM CaCl_2_, 100 mM NaCl, 1 mM EDTA, and 10 mM-β-mercaptoethanol) on ice for 20 min at 37°C for 5 min. After pre-run at 120V for 1 h, the electrophoresis was performed on an 8% native polyacrylamide gel in the Tris-borate-EDTA (TBE) buffer at 100 V for 1 h on ice. Bands were stained with ethidium bromide (0.5 µg/mL) and visualized with the molecular imager ChemiDoc XRS+ (Bio-Rad).

### Northern blot

Five micrograms total RNA isolated from the indicated bacteria was loaded onto a denaturing agarose gel containing 0.25 M formaldehyde and separated by electrophoresis. The gels were stained with ethidium bromide to visualize the 5S rRNA as a control. The fractionated RNA was transferred to a nylon membrane (Hybond N; Amersham). Northern blots were hybridized with sRNA1060 or sRNA1062-specific biotin-labeled probes, respectively. After hybridization and washing, the membrane was incubated with a streptavidin-HRP conjugate (GenScript). After washing, signals were detected by an ECL Plus kit (Millipore) and visualized by a Bio-Rad molecular imager (ChemiDocXRS).

## Data Availability

The RNA-seq raw data have been deposited in NCBI (PRJNA1070051). Other supporting data can be found in the supplemental material.

## SUPPLEMENTAL MATERIAL

Supplemental figures and tables mbio.01788-25-s0001.pdf Fig. S1 to S9; Tables S1 and S3.

Table S2 mbio.01788-25-s0002.xlsx BLAST of sRNA1060 and sRNA1062.

ASM does not own the copyrights to Supplemental Material that may be linked to, or accessed through, an article. The authors have granted ASM a non-exclusive, world-wide license to publish the Supplemental Material files. Please contact the corresponding author directly for reuse.
