# Supplementary material for: Pseudomonas aeruginosa lasR-deficient mutant contributes to bacterial virulence through enhancing the PhoB-mediated pathway in response to host environment
Source: mBio. 2025 Sep 4;16(10):e01788-25. doi: 10.1128/mbio.01788-25 (PMC12506140; doi:10.1128/mbio.01788-25)
Supplement: Supplemental figures and tables — Fig. S1 to S9; Tables S1 and S3. [file mbio.01788-25-s0001.pdf]

Fig. S1

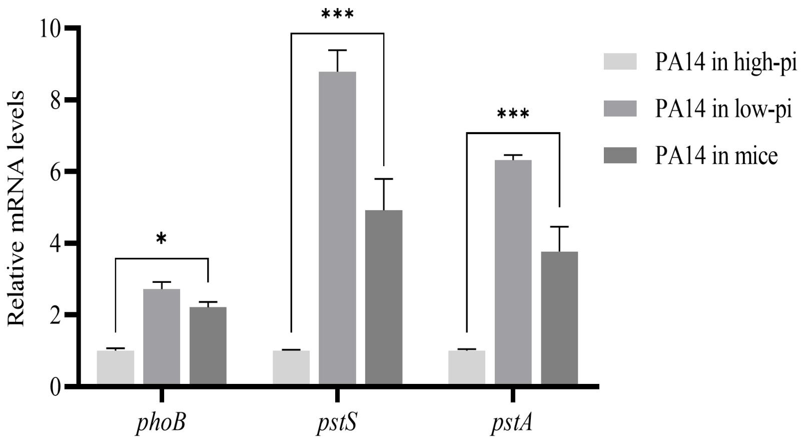

Figure S1. Relative mRNA levels of *phoB*, *pstS* and *pstA* of PA14 in the high, low phosphate media or *in vivo*. *, P<0.05; ***, P<0.001 by ANOVA. Data represent the mean ± standard deviation from three samples.

Fig. S2

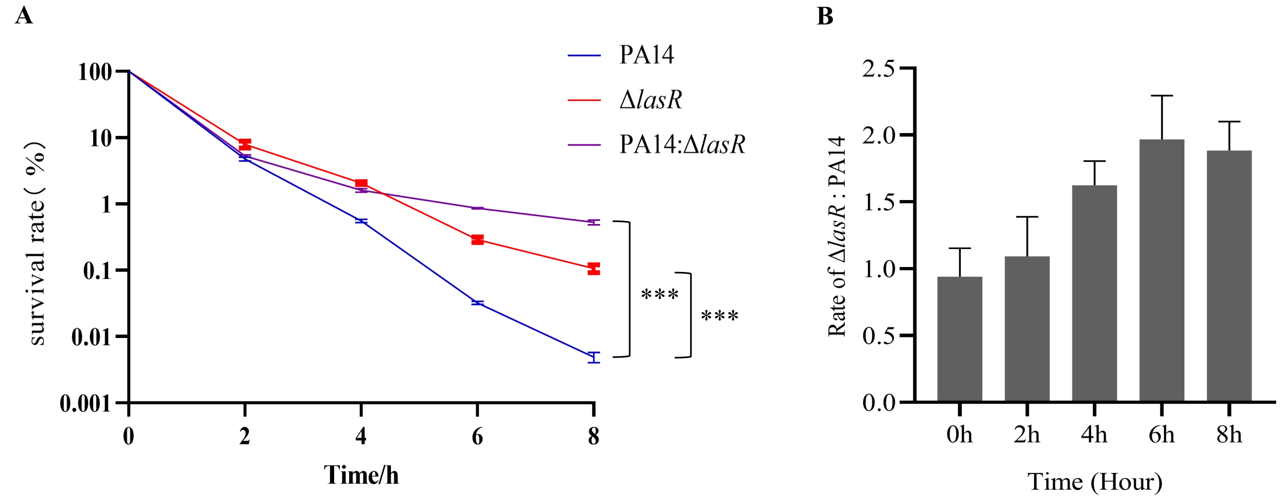

Figure S2. The time-kill assays of ceftazidime. PA14, the Δ*lasR* or the wild type-Δ*lasR* combination were grown in the low phosphate medium to an OD_{600} of 1.0 at 37 ° C and treated with 8 μg/ml ceftazidime. At indicted time points, the live bacterial numbers (A) and the ratios between the Δ*lasR* mutant and wild type PA14 in the combined population (B) were determined.

Fig. S3

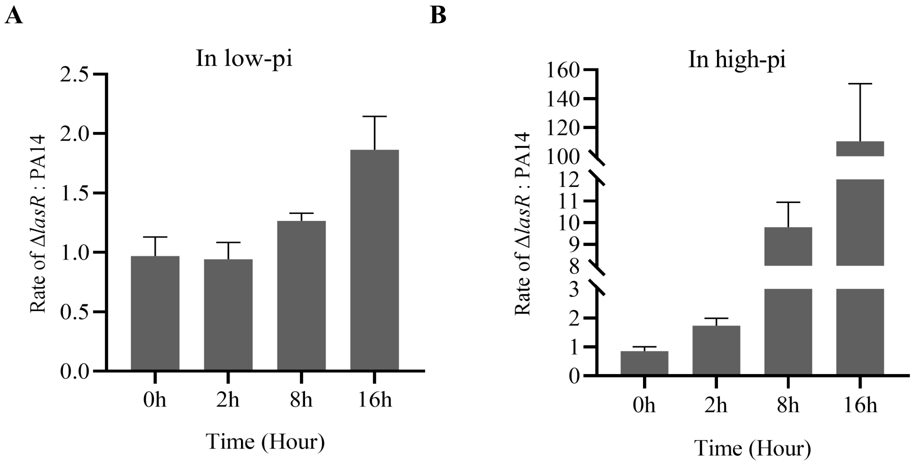

Figure S3. Wild type PA14 and the Δ*lasR* mutant were mixed at a ratio of 1:1 and grown in the low phosphate medium (A) or high phosphate medium (B). At the indicated time points, the ratios between wild type PA14 and the Δ*lasR* mutant were determined.

Fig. S4

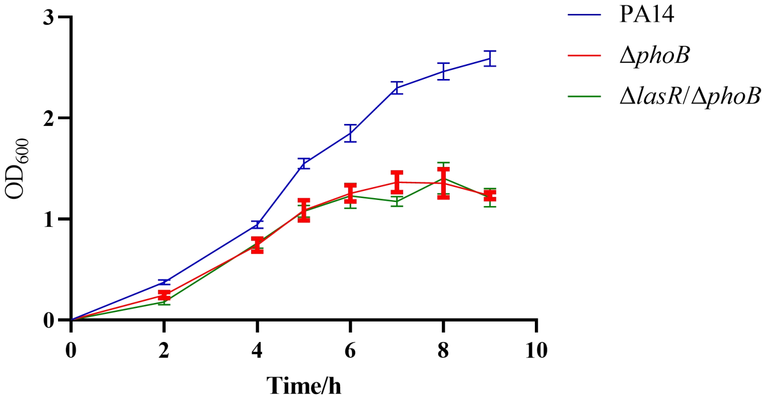

Figure S4. Bacterial growth in low phoaphate condition. PA14, the Δ*phoB* or the Δ*lasR/*Δ*phoB* growth were monitored by measuring OD_{600} for 9 h. Data represent the mean ± standard deviation of assays performed in triplicate and are representative of three independent experiments with similar results

Fig. S5

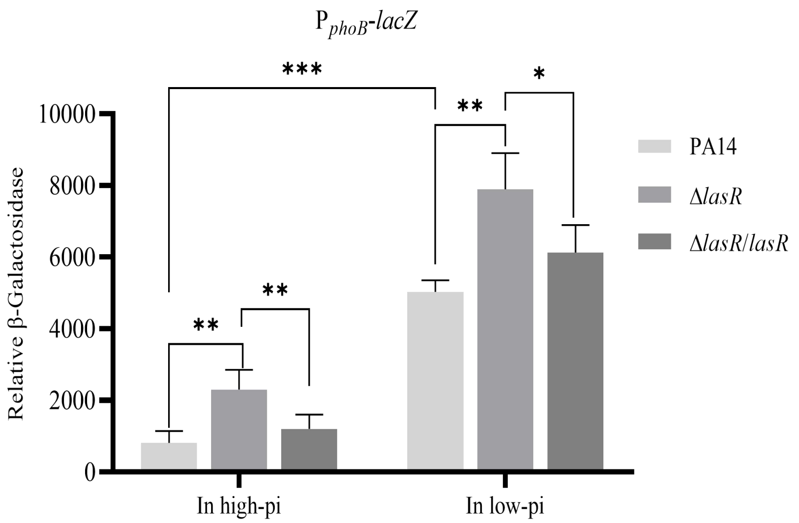

Figure S5. The wild type PA14, Δ*lasR* and Δ*lasR/lasR* carrying the P_{phoB}-*lacZ* transcriptional fusion were grown in the low or high phosphate medium to an OD_{600} of 1.0, followed by β-galactosidase assays. *, P<0.05; **, P<0.01; ***, P<0.001 by ANOVA.

Fig. S6

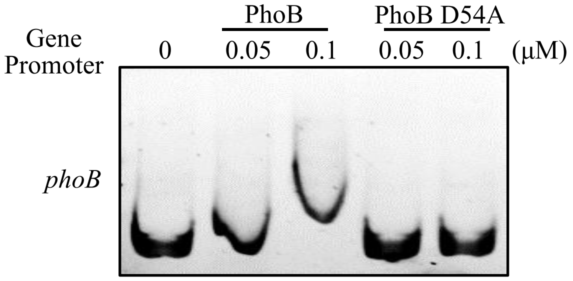

Figure S6. EMSA analysis of PhoB and PhoB D54A binding to the promoter regions of phoB.

Fig. S7

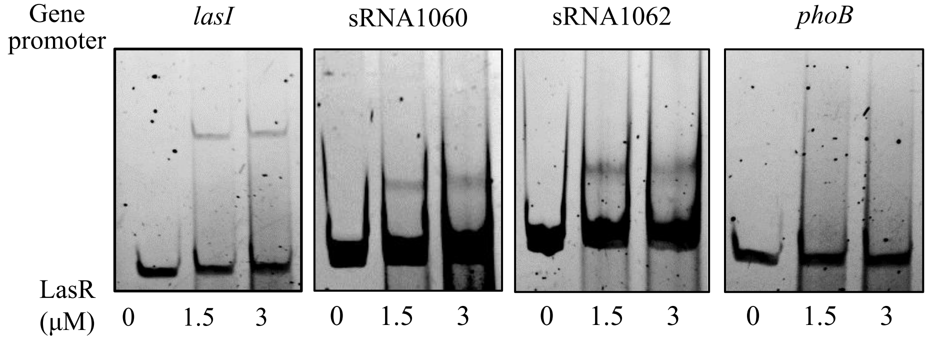

Figure S7. The binding of LasR protein to the promoter regions of sRNA 1060 and sRNA 1062 were examined by EMSA. The promoter regions of lasI and phoB were used as positive and negative controls, respectively.

Fig. S8

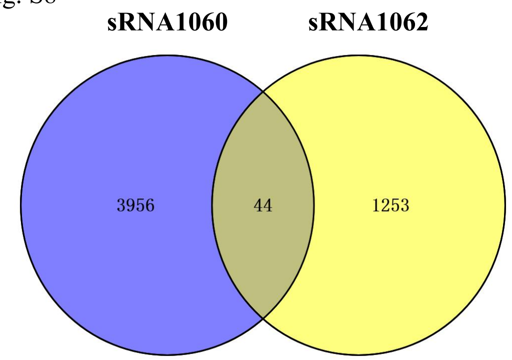

Figure S8. Venn diagram of sRNA1060 and sRNA1062 homologs in a total of 7906 *P. aeruginosa* genomes.

Fig. S9

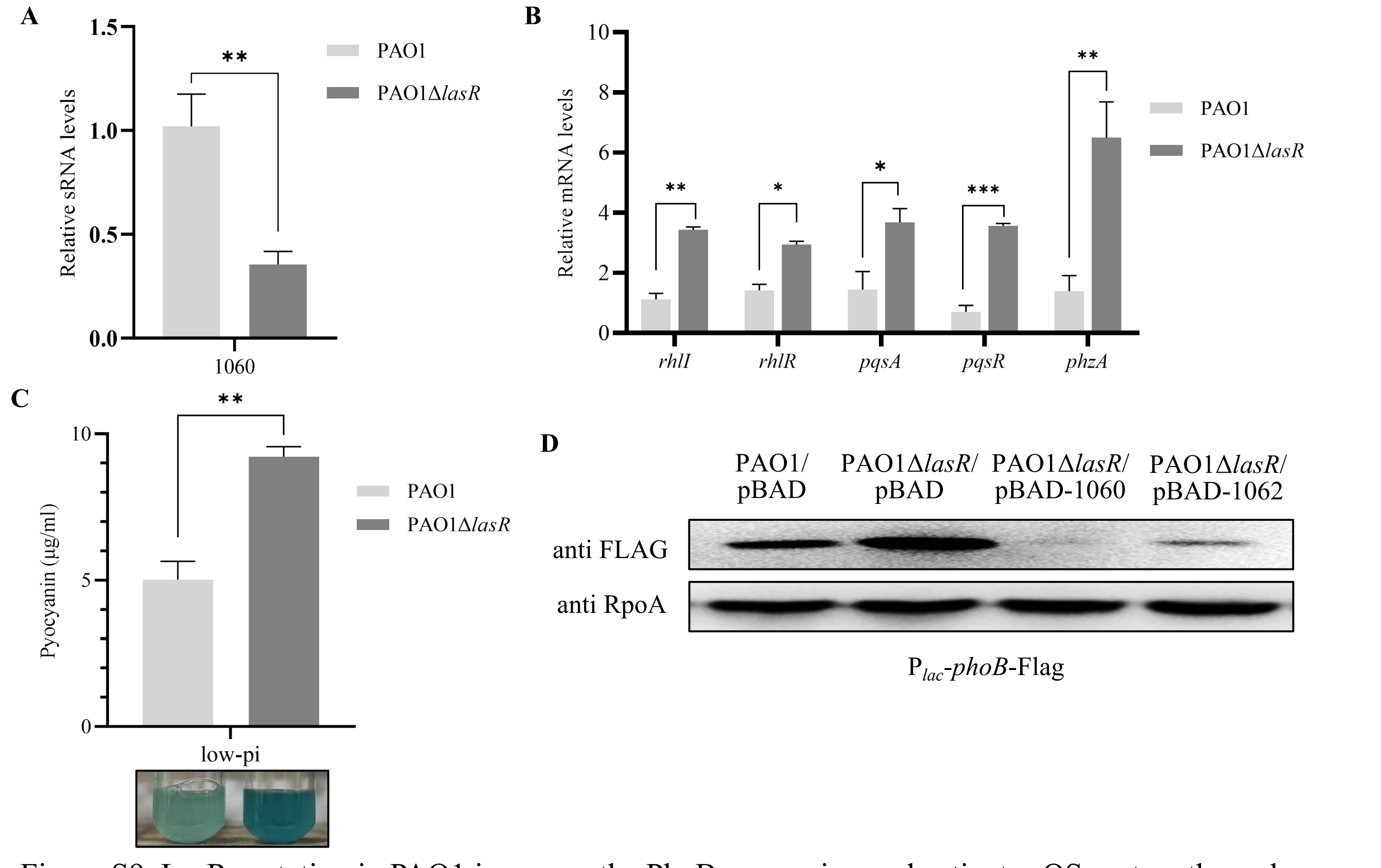

Figure S9. LasR mutation in PAO1 increases the PhoB expression and activates QS system through sRNA 1060. (A) Relative mRNA levels of *rhlI*, *rhlR*, *pqsA*, *pqsR* and *phzA* PAO1 and PAO1Δ*lasR* in the low phosphate medium. *, P<0.05; **, P<0.01; ***, P<0.001 by ANOVA. (B) Pyocyanin concentrations of overnight cultures of PAO1 and PAO1Δ*lasR* in the low phosphate medium. The final values were normalized by OD_{600} of each bacterial cultures. Data represent the mean ± standard deviation of assays performed in triplicate and are representative of three independent experiments with similar results. **, P < 0.01 by ANOVA. (C) Relative levels of sRNA1060 and sRNA1062 in PAO1 and PAO1Δ*lasR* mutant in LB media. **, P<0.01 by ANOVA. (D) sRNA1060, sRNA1062 was overexpressed in the PAO1Δ*lasR* mutant. Bacteria containing the P*lac*-phoB-Flag were grown to an OD_{600} of 1.0 in LB. The levels of PhoB-FLAG and RpoA were determined by western blot. Data shown are representative of three independent experiments with similar results.

Table S1. sRNAs differentially transcribed in transcriptome sequencing

PA14-HP: PA14 in high Pi medium; lasR-HP: Δ*lasR* in high Pi medium;PA14-LP: PA14 in low Pi medium; lasR-HP: Δ*lasR* in low Pi medium;

sRNA_ID	Start	End	Strand	Expression_PA14-HP	Expression_lasR-HP	Expression_PA14-LP	Expression_lasR-LP	Qvalue_PA14-HP-VS-lasR-HP	Qvalue_PA14-LP-VS-lasR-LP
sRNA1042	352447	352499	+	526	1203	670	616	0.086893116	1
sRNA1043	405240	405058	-	26	20	1845	1949	1	1
sRNA1044	425730	425968	+	1736	293	1709	1181	2.81E-05	1
sRNA1045	515997	515892	-	852	65	54	85	6.72E-50	1
sRNA1046	642276	642471	+	1209	1957	765	1077	1	1
sRNA1047	755065	755132	+	990	264	478	250	0.004748795	1
sRNA1048	799927	800008	+	1436	1700	371	387	1	1
sRNA1049	813249	813457	+	319	261	2505	7295	1	1
sRNA1050	1608425	1608331	-	1347	1144	1031	1064	1	1
sRNA1051	1649520	1649584	+	1005	38	10563	4542	3.41E-188	1
sRNA1052	1650868	1650989	+	440	64	2648	1234	3.71E-11	1
sRNA1053	1675920	1675819	-	225	585	2234	1572	0.030910272	1
sRNA1054	1750183	1750249	+	0	4	3377	4264	1	1
sRNA1055	2135028	2134933	-	1405	218	1576	2740	6.66E-10	1
sRNA1056	2275339	2275185	-	1358	65	669	54	1.48E-139	3.07E-29
sRNA1057	2451322	2451127	-	1311	57	621	869	1.31E-178	1
sRNA1058	2678005	2677736	-	2353	1442	1533	2233	1	1
sRNA1059	2925612	2925548	-	1362	56	1421	556	1.61E-148	0.528990735
sRNA1060	3103908	3103992	+	1606	12	1686	965	0	1
sRNA1061	3831504	3831428	-	1397	261	760	465	1.77E-06	1
sRNA1062	4133017	4133234	+	1344	626	412	496	0.038737773	1
sRNA1063	4464956	4464891	-	925	2044	1172	1030	0.117964708	1
sRNA1064	4562753	4562676	-	1230	267	462	410	1.01E-04	1
sRNA1065	4571605	4571788	+	1800	389	1511	2229	0.004820966	1
sRNA1066	4844626	4844690	+	1647	170	1470	1783	1.24E-23	1
sRNA1067	5044877	5044729	-	429	207	2801	1283	0.370163884	1
sRNA1068	5288206	5288410	+	1195	2118	1215	1360	1	1
sRNA1069	5291080	5291131	+	679	1818	1886	1572	0.025227175	1
sRNA1070	5502056	5501979	-	690	1744	1071	577	0.048667117	1
sRNA1071	5556301	5556194	-	1519	176	172	360	2.61E-19	0.541157146
sRNA1072	5597542	5597487	-	1014	262	495	309	0.004529601	1
sRNA1073	5778052	5778299	+	70	1244	80	258	2.11E-170	0.013255853
sRNA1074	6017261	6017052	-	282	157	1289	173	0.672549844	4.48E-13
sRNA1075	6306129	6306035	-	123	129	2539	2961	1	1
sRNA1076	6394094	6393989	-	16	18	4600	4389	1	1

Table S3. Bacterial strains, plasmids and primers used in this study.

Strain/ Plasmid /Primer	Description	Source (Reference)
P. aeruginosa		
PA14	Wild type strain of *Pseudomonas aeruginosa*	(1)
Δ*lasR*	PA14 deleted of *lasR*	(2)
Δ*phoB*	PA14 deleted of *phoB*	(2)
Δ*lasR*/Δ*phoB*	PA14 deleted of *lasR* and *phoB*	This study
Δ*lasR*/*lasR*	PA14 deleted of *lasR* with plasmid pUC18T-mini Tn7T-Tc-*lasR*; Tc^r	This study
E. coli		
BL21/pET28a-*phoB*	PhoB protein expression strain; Kn^r	This study
BL21/pET28a-*phoB* D54A	PhoB protein with muatation D54A expression strain; Kn^r	This study
BL21/pET28a-*lasR*	LasR protein expression strain; Kn^r	This study
**Plasmid**		
pUC18T-mini Tn7T-Tc	Insertion vector, Tc^r	(3)
pMMB	Expression vector with *tac* promoter; Ap^r	(4)
pMMB-pBAD	Expression vector with pBAD promoter; Ap^r	This study
pMMB-AsR *phoB*	Clonging *phoB* antisense RNA on pMMB; Ap^r	(2)
pUC18T-mini Tn7T-Tc-*lacR*	Cloning of promoter and *lacR* on pUC18T-mini Tn7T-Tc, Tc^r	This study
pUC18T-mini Tn7T-Tc-P_{phoB}-*phoB*-FLAG	Cloning of *phoB* promoter and *phoB* fused with FLAG probe DNA on pUC18T-mini Tn7T-Tc, Tc^r	This study
pUC18T-mini Tn7T-Tc-P_{lac}-*phoB*-FLAG	Cloning of *lac* promoter and *phoB* fused with FLAG probe DNA on pUC18T-mini Tn7T-Tc, Tc^r	This study
pBAD-1044	Cloning of sRNA1044 on pBAD, Ap^r	This study
pBAD-1051	Cloning of sRNA1051 on pBAD, Ap^r	This study
pBAD-1052	Cloning of sRNA1052 on pBAD, Ap^r	This study
pBAD-1056	Cloning of sRNA1056 on pBAD, Ap^r	This study
pBAD-1059	Cloning of sRNA1059 on pBAD, Ap^r	This study
pBAD-1060	Cloning of sRNA1060 on pBAD, Ap^r	This study
pBAD-1062	Cloning of sRNA1062 on pBAD, Ap^r	This study
pBAD-1064	Cloning of sRNA1064 on pBAD, Ap^r	This study
pBAD-1071	Cloning of sRNA1071 on pBAD, Ap^r	This study
pBAD-1074	Cloning of sRNA1074 on pBAD, Ap^r	This study
pBAD-1060MUT	Cloning of sRNA1060MUT on pBAD, Ap^r	This study
pBAD-1062MUT	Cloning of sRNA1062MUT on pBAD, Ap^r	This study
**Primer**	**Sequence (5'→3')**	**Function**
comlasRF	CGCGGATCCGTGGTGGTGTGCGAGATG	LasR cloning to pUC18T-mini Tn7T
comlasRR	CGAGCTCCTGAGAGGCAAGATCAGAGAGTA	LasR cloning to pUC18T-mini Tn7T
EX *phoB* F	CCCTCGAGGCTCTTGGTGGAGAAACGATAG	*phoB* expression
EX *phoB* R	AGGCAAGACCATGGTTGGCAAG	*phoB* expression
phoB D54A F	ACCTGATCCTGCTCGCCTGGATGCTC	PhoB point mutation

phoB D54A R	CGGGGAGCATCCAGGCGAGCAGGATC	PhoB point mutation
EX-*lasR*F	CGCCATGGCCTTGGTTGACGGTT	Expression of LasR
EX-*lasR*R	CCCTCGAGAGTAATAAGACCCAAATTAACG	Expression of LasR
rpsL-RT-F	GTAAGGTATGCCGTGTACG	RT-PCR
rpsL-RT-R	CACTACGCTGTGCTCTTG	RT-PCR
pcrV-RT-F	GGTACTCCGCGAAGTCCTGC	RT-PCR
pcrV-RT-R	GTCGAGCAGCGCCTTGCG	RT-PCR
aprA-RT-F	AACAGGCGTCTTGGCAGAAA	RT-PCR
aprA-RT-R	TGAACTTGCCCAGCGAGTAGAT	RT-PCR
rhlI- RT-F	CCATCCGCAAACCCGCTACATCG	RT-PCR
rhlI- RT-R	CCGACGGATCGCTCGGCG	RT-PCR
rhlR- RT-F	CGTGGATCCGGCGATCCTC	RT-PCR
rhlR- RT-R	GCTGAGCAAATTGTTCG	RT-PCR
pqsA- RT-F	TCCCGATACCGCCGTTT	RT-PCR
pqsA- RT-R	AGCGAAGGTGAGTCGTTCAAC	RT-PCR
pqsR- RT-F	CAGCACCCGTTGTGCAATG	RT-PCR
pqsR- RT-R	CAGACGCAGCATGTCGTCG	RT-PCR
phzA- RT-F	AACGTGCGGATCTTCGAG	RT-PCR
phzA- RT-R	CCGTTCTCGAGTTCGAAG	RT-PCR
plcH- RT-F	GAAAGCGGAGAGGAACTGGG	RT-PCR
plcH- RT-R	CGTGGAACTCGCGGAAGAA	RT-PCR
plcN- RT-F	AACTTCGCCAACCCGAAC	RT-PCR
plcN- RT-R	TTCCTGCTTGGGCATGGCGG	RT-PCR
phoB- RT-F	CCGCTGTTCCGTCATTCC	RT-PCR
phoB- RT-R	GTCGTCACCGTGCTTTTCG	RT-PCR
pstA- RT-F	TGCAGGACATCAAGCGTCC	RT-PCR
pstA- RT-R	CTTCTGCAGTTCGCCTAC	RT-PCR
pstS- RT-F	AGCAAGCAGGACGTGAAGAC	RT-PCR
pstS- RT-R	GACGTTCGGACGGAAG	RT-PCR
1044- RT-F	GCTGCGTCGCTGGATTTC	RT-PCR
1044- RT-R	GCCCGCCTTTCGTTGG	RT-PCR
1051- RT-F	ACCCTTGACCTGCGAAGACC	RT-PCR
1051- RT-R	GGCTACTCCGTGCGTTATGC	RT-PCR
1052- RT-F	GGTGCTGGCATAACAGATAGGG	RT-PCR
1052- RT-R	CGATAAAATGCATCACAGCAGAAT	RT-PCR
1056- RT-F	TCCCAGCCCCGACCAA	RT-PCR
1056- RT-R	GCGGAAGAATTGCTGATAGCTC	RT-PCR
1059- RT-F	CCGCTCTGGCGTCGTG	RT-PCR
1059- RT-R	TTAAGCTGGCAGAACTGACAGG	RT-PCR
1060- RT-F	CGGTTCGTCGGACAGCC	RT-PCR
1060- RT-R	TTACCGCTCCTCGGATGAA	RT-PCR
1062- RT-F	TAGAGTTGGCCTTCCATGGC	RT-PCR
1062- RT-R	GAAAGTTACTGCTTCCGCTGTG	RT-PCR
1064- RT-F	TAGATAGCGCCCGAGTGTTTC	RT-PCR

1064- RT-R	TCACTGACCGACGGGAGGT	RT-PCR
1071- RT-F	CTGGAGCCGCGTATCGC	RT-PCR
1071- RT-R	TCTCCTCATCAGGCTAATCACG	RT-PCR
1074- RT-F	CGCCTGCTCAAGAAACGAC	RT-PCR
1074- RT-R	CGACGCCCTGTGGACAAGT	RT-PCR
phoBflagF-EcoRI	CGGAATTCCTGCACAACCACTGGG	Fusion PhoB with FLAG
phoBflagR-SacI	CGAGCTCACTTGTCATCGTCGTCCTTGTAATCGCTCTTGGTGGAGAAACGATAG	Fusion PhoB with FLAG
Plac-F-SmaI	TCCCCCGGGTAGGCACCCCAGGCTTTAC	Fusion lac promoter with *phoB*
phoB-ATG-F-EcoRI	CGGAATTCATGGTTGGCAAGACAATCCTC	Fusion lac promoter with *phoB*
EM-rhlRF	GCCTGTCGGCGTTTCATG	EMSA of rhlR
EM-rhlRR	TAAGCCCTGATCGATAAAATGC	EMSA of rhlR
EM-pqsRF	GTTTCTTAGAACCGTCCCTAGCTC	EMSA of pqsR
EM-pqsRR	ATTCCTTTTATTGGGTGGCG	EMSA of pqsR
EM-phoBF	GTCCTGGATGAAGGTATAAAGGG	EMSA of phoB
EM-phoBR	CGTCATCAACGATGAGGATTGT	EMSA of phoB
EM1060F	GCGAGGTCGCCACCGAGCTGA	EMSA of sRNA1060
EM1060R	CGAACGCCTCGAGACACAGGCG	EMSA of sRNA1060
EM1062F	TCAAGCAACTGGCGGCAGCGAACATC	EMSA of sRNA1062
EM1062R	CGTCTGTCTCCGCACCGATAGGGAA	EMSA of sRNA1062
SacI-PbadF	CAAGAGCTCTTATGACAACTTGACGG	Clone pBAD to pMMB
Pbad-XbaIR	GCTCTAGAAACAGTAGAGAGTTGCGATAAAAAGCG	Clone pBAD to pMMB
OE1044F	CGGCTAGCTGTCGTTATGGCGGACCTGGG	Over expression of sRNA1044
OE1044TR	CCAAGCTTCGATATGGTTGGTGGTGACGCTG	Over expression of sRNA1044
OE1051F	GCTCTAGACCCTTGACCTGCGAAGACCCG	Over expression of sRNA1051
OE1051TR	CCCAAGCTTGCAGAGGCTCACGCGGTG	Over expression of sRNA1051
OE1052F	GCTCTAGAGGTGCTGGCATAACAGATAGGGTTG	Over expression of sRNA1052
OE1052TR	CCAAGCTTTTCCAGGACGGCGAACACG	Over expression of sRNA1052
OE1056F	GCTCTAGACCATCCTCCCAGCCCCGA	Over expression of sRNA1056
OE1056TR	CCCAAGCTTCCTTGTGAGGCGATGATCGATAAG	Over expression of sRNA1056
OE1059F	GCTCTAGAGCGCCGCTCTGGCGTCGTGTT	Over expression of

		sRNA1059
OE1059TR	CCCAAGCTTCGTCCAGAAGGTTTCGCGTTATC	Over expression of sRNA1059
OE1060F	GCTCTAGATCTCGAGGCGTTCGCGGCG	Over expression of sRNA1060
OE1060R	CCCAAGCTTACCGCTCCTCGGATGAAGGTAGGAAAC	Over expression of sRNA1060
OE1062F	GCTCTAGAGTGCGGAGACAGACGGAC	Over expression of sRNA1062
OE1062TR	CCAAGCTTCGACGGCGTTTCAAAGGTTTCACAC	Over expression of sRNA1062
OE1064F	GCTCTAGATCGATAGATAGCGCCCGAGTGTTTC	Over expression of sRNA1064
OE1064TR	CCAAGCTTCATCCCTCACCTCCAAAACGACG	Over expression of sRNA1064
OE1071F	GCTCTAGAGGAAGGGCGCGAGGCC	Over expression of sRNA1071
OE1071TR	CCAAGCTTGAGAACCGGCTTGACCTGATAATGAG	Over expression of sRNA1071
OE1074F	GCTCTAGACCTTTTCCGGAGAAGAGGCCAC	Over expression of sRNA1074
OE1074TR	CCCAAGCTTGTCAGCGTAGTGCCGCGC	Over expression of sRNA1074
mut1060F	GTTCGCGGCGAGGTGCCAGCGGTT	Mutation of sRNA1060
mut1060R	GACGAACCGCTGGCACCTCGCCG	Mutation of sRNA1060
mut1062F	CGTCATATGCCACAAAAGCCGGGGCT	Mutation of sRNA1062
mut1062R	TTCCAGCCCCGGCTTTTGTGGCATATG	Mutation of sRNA1062
1060biotin	ATCCGGAGGGGCTGTCCGACGAACCGC	Add a biotin to sRNA1060 probe for northern blot
1062biotin	TAGATACTTTATGAGGTAAAGCTAAAATCCACC	Add a biotin to sRNA1062 probe for northern blot

References

1. N. T. Liberati *et al.*, An ordered, nonredundant library of Pseudomonas aeruginosa strain PA14 transposon insertion mutants. *Proceedings of the National Academy of Sciences of the United States of America* **103**, 2833-2838 (2006).
2. X. Zhao *et al.*, PitA Controls the H2- and H3-T6SSs through PhoB in Pseudomonas

aeruginosa. *Appl Environ Microbiol* **89**, e0209422 (2023).
3. K. H. Choi, H. P. Schweizer, mini-Tn7 insertion in bacteria with single attTn7 sites: example Pseudomonas aeruginosa. *Nature protocols* **1**, 153-161 (2006).
4. J. P. Fürste *et al.*, Molecular cloning of the plasmid RP4 primase region in a multi-host-range tacP expression vector. *Gene* **48**, 119-131 (1986).
